# Solanaceae—A Model for Linking Genomics With Biodiversity

**DOI:** 10.1002/cfg.393

**Published:** 2004-04

**Authors:** Sandra Knapp, Lynn Bohs, Michael Nee, David M. Spooner

**Affiliations:** 1 Department of Botany Natural History Museum Cromwell Road London SW7 5BD UK; 2 Department of Biology 257 South 1400 East University of Utah Salt Lake City UT 84112 USA; 3 Herbarium The New York Botanical Garden Bronx River Parkway at Fordham Road Bronx NY 10458 USA; 4 USDA, Agricultural Research Service Department of Horticulture University of Wisconsin 1575 Linden Drive Madison WI 53706-1590 USA

## Abstract

Recent progress in understanding the phylogeny of the economically important plant family Solanaceae makes this an ideal time to develop models for linking the new data on plant genomics with the huge diversity of naturally occurring species in the family. Phylogenetics provides the framework with which to investigate these linkages but, critically, good species-level descriptive resources for the Solanaceae community are currently missing. Phylogeny in the family as a whole is briefly reviewed, and the new NSF Planetary Biodiversity Inventories project ‘PBI: Solanum—a worldwide treatment’ is described. The aims of this project are to provide species-level information across the global scope of the genus *Solanum* and to make this available over the Internet. The project is in its infancy, but will make available nomenclatural information, descriptions, keys and illustrative material for all of the approximately 1500 species of *Solanum*. With this project, the opportunity of linking valid, up-to-date taxonomic information about wild species of *Solanum* with the genomic information being generated about the economically important species of the genus (potato, tomato and eggplant) can be realized. The phylogenetic framework in which the PBI project is set is also of enormous potential benefit to other workers on *Solanum*. The community of biologists working with Solanaceae has a unique opportunity to effectively link genomics and taxonomy for better understanding of this important family, taking plant biology to a new level for the next century.


**Comparative and Functional Genomics**
*Comp Funct Genom* 2004; **5**: 285–291.
Published online in Wiley InterScience (www.interscience.wiley.com). **DOI:** 10.1002/cfg.393

**Conference Review**

### Solanaceae — a model for linking genomics with biodiversity

Sandra Knapp[1]*, Lynn Bohs[2], Michael Nee[3] and David M. Spooner[4]
[1] *Department of Botany, Natural History Museum, Cromwell Road, London SW7 5BD, UK*
[2] *Department of Biology, 257 South 1400 East, University of Utah, Salt Lake City, UT 84112, USA*
[3] *Herbarium, The New York Botanical Garden, Bronx River Parkway at Fordham Road, Bronx, NY 10458, USA*
[4] *USDA, Agricultural Research Service, Department of Horticulture, University of Wisconsin, 1575 Linden Drive, Madison, WI 53706-1590 USA*

**Correspondence to: Sandra Knapp, Department of Botany, Natural History Museum, Cromwell Road, London, SW7 5BD, UK. E-mail: s.knapp@nhm.ac.uk*

Received: 28 January 2004
Accepted: 6 February 2004

### Abstract

**Recent progress in understanding the phylogeny of the economically important plant family Solanaceae makes this an ideal time to develop models for linking the new data on plant genomics with the huge diversity of naturally occurring species in the family. Phylogenetics provides the framework with which to investigate these linkages but, critically, good species-level descriptive resources for the Solanaceae community are currently missing. Phylogeny in the family as a whole is briefly reviewed, and the new NSF Planetary Biodiversity Inventories project 'PBI: *Solanum* — a worldwide treatment' is described. The aims of this project are to provide species-level information across the global scope of the genus *Solanum* and to make this available over the Internet. The project is in its infancy, but will make available nomenclatural information, descriptions, keys and illustrative material for all of the approximately 1500 species of *Solanum*. With this project, the opportunity of linking valid, up-to-date taxonomic information about wild species of *Solanum* with the genomic information being generated about the economically important species of the genus (potato, tomato and eggplant) can be realized. The phylogenetic framework in which the PBI project is set is also of enormous potential benefit to other workers on *Solanum*. The community of biologists working with Solanaceae has a unique opportunity to effectively link genomics and taxonomy for better understanding of this important family, taking plant biology to a new level for the next century.** **Copyright © 2004 John Wiley & Sons, Ltd.**

**Keywords:** **Solanaceae; phylogeny; taxonomy; genomics; potato; tomato; tobacco; *Solanum***

### Introduction

Among angiosperm families, the Solanaceae rank as one of the most important to human beings. Species of the family are used for food (e.g. *Solanum tuberosum* L., the potato; *S. lycopersicum* L., the tomato; *S. melongena* L., the eggplant), drugs (e.g. *Nicotiana tabacum* L. and *N. rustica* L., tobacco; *Atropa belladonna* L., deadly nightshade; *Mandragora officinarum* L., mandrake; *Duboisia* spp., sources of commercial alkaloids) and as ornamentals (e.g. *Petunia hybrida* hort., petunia; *Salpiglossis sinuata* Ruiz & Pavón, velvet tongue; *Schizanthus pinnatus* Ruiz & Pavón, butterfly flower: Figure 1A). The family is a medium-sized one with approximately 90 genera and 3000–4000 species, almost of half of which are in the large and diverse genus *Solanum*. This hyperdiversity in one genus is unusual in angiosperms, making *Solanum* interesting from an evolutionary standpoint as well as for its usefulness to humans. Members of the Solanaceae are extremely diverse; in terms of habit, ranging from trees to small annual herbs; in habitat, from deserts to the wettest tropical rain forests;

Copyright © 2004 John Wiley & Sons, Ltd.

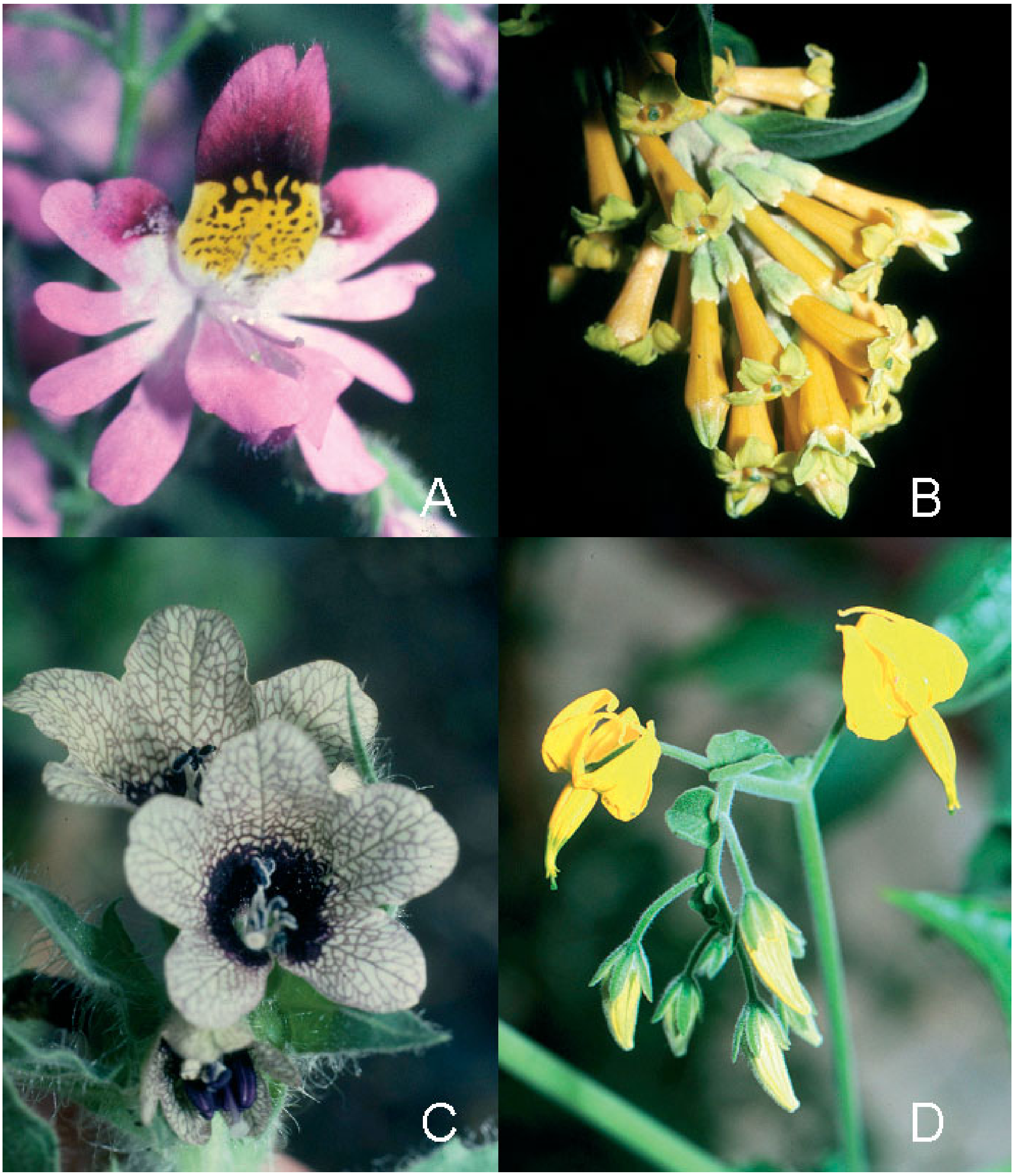

**Figure 1.** Examples of flower shape variation in the Solanaceae (all photos by S. Knapp); see Figure 2 for clades in the Solanaceae. (A) Highly zygomorphic flowers of *Schizanthus pinnatus* Ruiz & Pavón — *Schizanthus* (cult. Botanical Garden, University of Nijmegen, The Netherlands; Accession No. 924750085). (B) Tubular flowers of *Cestrum psittacinum* Stapf — *Cestrum* clade (cult. Chelsea Physic Garden, London, UK). (C) Slightly zygomorphic flowers of *Hyoscyamus niger* L. — *Hyoscyamus* clade (cult. Chelsea Physic Garden, London, UK). (D) Nearly actinomorphic flowers of *Solanum peruvianum* L. — *Solanum* clade (cult. Botanical Garden, University of Nijmegen, The Netherlands; Accession No. 974750064)

Copyright © 2004 John Wiley & Sons, Ltd.

and in morphology, with astounding variation in many characters of both flowers and fruits (see Figure 1A–D; for more photographs of morphological variation in the Solanaceae, see [14–16]). Both generic and species level diversity in the family is concentrated in the Andes of South America, and the family has a classic Gondwanan distribution [24]. The last complete taxonomic treatment at the species level for the entire family was made over a century ago [5] and since then work has been concentrated in smaller generic level groups or in regional floristic treatments.

### What is taxonomy?

Taxonomy is critical to the study of biodiversity [7], and thus it is worth taking the space here to describe just what taxonomy is for those who perhaps do not use it often in their day-to-day work. Some people define taxonomy and systematics as two completely separate aspects of the science, but we prefer to focus on what we consider the three essential components of the science. With that in mind, we will use the two terms interchangeably, but will focus on just what the science is and, more importantly, how its application to the family Solanaceae can help link genomics with biodiversity.

Taxonomy can be thought of as three interlocking spheres of endeavour — *description, identification* and *phylogeny*. Each one of these components is enriched by the other two and without all three the science is incomplete. Phylogeny is the study of the interrelationships of organisms — how species, genera and families are related to one another. We know that all of life is related in some way; phylogeny is the assembly of the tree of life. Today, phylogeny is done using the principles of cladistics, first laid out by the German biologist Willi Hennig ([11]; see also other references). Hennig's methodology involves using shared, derived characteristics (synapomorphies) to group species into monophyletic *clades*, sets of taxa all descended from a common ancestor. That lions and humans share a backbone, four limbs and fur unites the two in a clade, an inclusive group. It does not imply that humans have descended from lions, or vice versa — it only states that lions and humans share characters not shared with fish, for example, or insects. Characters can be physical characteristics of the organism, such as feathers, fur or flower colour, or can be a sequence of bases in DNA. Today, much phylogeny is done using these latter molecular characters. A phylogenetic tree is a pictoral depiction of nested sets of shared characteristics and is a powerful framework for interpretation of patterns in nature [4,22]. It is not, however, the last word. Each phylogeny is a hypothesis about the relationships of the organism under study, and taxonomists use what Hennig called 'reciprocal illumination' to solve problems and investigate apparent conflicts further [13]. More data or a different interpretation of the same data can cause us to modify the hypothesis, and thus our ideas of how organisms are related. The construction of phylogenetic trees for many groups of organisms has revolutionized the field of systematics and has led to new interpretations of how groups are related, as well as making character analysis possible in an evolutionary framework.

### Phylogenetic work in the Solanaceae

Recent work using a variety of datasets derived from plastid DNA [6,18–20] has revealed interesting phylogenetic patterns in the Solanaceae. For example, *Nicotiana* (the tobaccos) and the members of an endemic Australian tribe, the Anthocercidae, were shown to form a well-supported group within a larger monophyletic clade of taxa whose base chromosome number is $x = 12$ [20]. The two groups had not been thought closely related previously, and both of the groups in the '$x = 12$ clade' had been considered to belong to different subfamilies. Mapping morphological characters such as flower shape (zygomorphic vs. actinomorphic) or fruit type (berries vs. capsules) onto a phylogeny derived from molecular characters (see Figure 2; [15,16,20]) can reveal questions of interest to the study of evolution and development or to ecology. Zygomorphic flowers have apparently evolved several times independently in different clades of the family and zygomorphy appears to be whorl-specific. This leads to the questions: do the same genes control these transitions, and how do they operate in the family? Work with *Petunia* and *Schizanthus* is beginning to unravel the answers to some of these questions ([3,17,25]; Karine Coenen, personal communication), but a phylogenetic framework is critical to the framing of future

Copyright © 2004 John Wiley & Sons, Ltd. 

large-scale hypotheses. As another example, the possession of baccate fruit (fleshy berries) appears to be a synapomorphy of a large monophyletic clade that includes the familiar tomatoes and eggplants. But using the phylogenetic framework reveals that there is homoplasy (parallel evolution or reversal) in both fleshy berries (found in *Cestrum* and *Duboisia* of the *Anthocercis* clade) in the largely capsular-fruited group and in the possession of dehiscent fruits (e.g. *Hyoscyamus* clade, *Datura*) in members of the berry-fruited clade. Could ecological factors be important in this pattern? Only by considering characters in a phylogenetic framework can we formulate such questions. Looking at several characters mapped onto the molecular phylogeny (see Figure 2), we can see that Solanaceae are truly 'paradoxical plants' [10]; the basal clades contain species of annual habit with strongly zygomorphic flowers, exactly the opposite to the 'trends' in the rest of the angiosperms [23].

The two other faces of taxonomy are just as important as phylogeny, but have slightly different products. Identification is perhaps the easiest to see as being of immediately practical use — it is obviously critical to be able to identify the organisms of interest in order to construct a phylogeny, or to sequence the same species in two different experiments, or to use the same species for breeding purposes. Reliable identification is one of the things that make biology repeatable. Identification also plays a critical role in ecological studies and in conservation. We can only discover if biodiversity is in decline if we can reliably identify its components over and over again, allowing monitoring to determine trends.

### The PBI: *Solanum* project

Underpinning both phylogeny and identification is description, the Cinderella of the world of taxonomy and systematics. Description is what many people would call taxonomy — the naming of species. Giving things names allows we humans to talk about them, but description in the taxonomic sense is much more than just the coining of a new name. A good taxonomic description is just that, a description of the organism — what it looks like, where it lives, sometimes even the base sequence of portions of its DNA, and perhaps, in the future, its entire genome. The characters used in both phylogeny and identification are parts of a description and are every bit as important as the name itself. In fact, the name is really just a shorthand way of accessing the information contained in the description itself, just as a person's name is easier to use than repeating a physical description each time one wants to refer to an individual. As mentioned earlier, the last complete descriptive taxonomic treatment of the family Solanaceae was done in the nineteenth century, using the techniques of that century. Today, we have the power of the Internet at our disposal, providing new opportunities and challenges (see [8]).

Through the Planetary Biodiversity Inventories programme, the National Science Foundation of the USA (NSF) is providing funds for the completion of digital, Internet-available descriptive monographs of key groups (see **http://www.nsf.gov/bio/pubs/awards/pbify03.htm** and **http://clade.acnatsci.org/allcatfish/ACSI/idx_pages/PBI_index.html**). One of the groups selected is the genus *Solanum*, representing approximately half of the species diversity of the family Solanaceae. The project 'PBI *Solanum*: a worldwide treatment' has three broad objectives: (a) to provide a global species-level taxonomic treatment for *Solanum*; (b) to make this treatment, with specimens, descriptions, keys and illustrative material, available on the Internet; and (c) to link the taxonomy of wild species with emerging genomics datasets. The project began in January 2004 and will ultimately include descriptions of all of the approximately 1500 species of wild solanums, including the relatives of the tomato (*S. lycopersicum*), potato (*S. tuberosum*) and eggplant (*S. melongena*) and a host of other minor crops and their relatives (see [10] for some of these emerging crops). This programme, coupled with the newly proposed International Solanaceae Genome Project (SOL; see **http://www.sgn.cornell.edu**), gives biologists working with the Solanaceae, and more specifically with *Solanum*, the unique opportunity to truly bring plant biology to a new level. The first of two big questions posed in the recent Solanaceae 'White Paper' (see the Solanaceae Genomics Network site at **http://www.sgn.cornell.edu/solanaceaeproject/**), 'How can a common set of genes/proteins give rise to a wide range of morphologically and ecologically distinct organisms that occupy our planet?', can only begin to be

Copyright © 2004 John Wiley & Sons, Ltd.

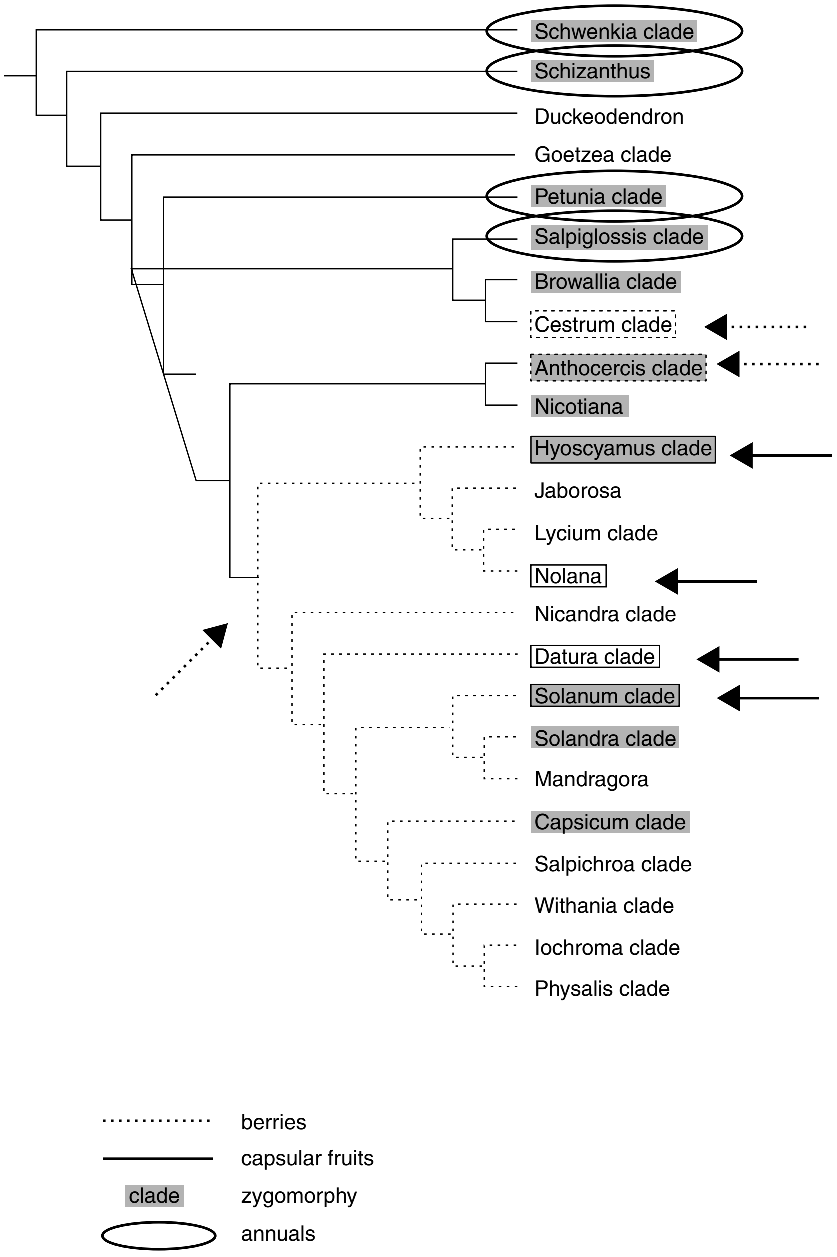

**Figure 2.** A phylogenetic tree of the Solanaceae based largely upon molecular data [5,18], showing the distribution of a selection of morphological characters in the family. This diagram is only a heuristic tool for exploring these distributions and should not be taken as the phylogeny of the family. Those interested in the details of the analyses are referred to the original literature. Genera belonging to each of the clades used here can be found in Table 2 of Knapp [16]. Dotted lines indicate the monophyletic group possessing baccate fruits (berries); clades with solid lines are those with capsular fruits. Homoplasy (see text) in possession of berries (*Cestrum, Duboisia* of the *Anthocercis* clade) is indicated with dotted arrows, while putative parallel evolution of dehiscent fruits (e.g. *Hyoscyamus* clade, *Datura*, some species of *Solanum, Oryctes* of the *Physalis* clade. etc.) is indicated with solid arrows (see [16] for further details of both fruit types and clade composition). Clades filled with grey are those with zygomorphic flowers and those ringed in black are those where the predominant growth form is annual

Copyright © 2004 John Wiley & Sons, Ltd. 

answered by bringing together the forces of taxonomy, in all three of its faces, and genomics.

How can we bring these two very different worlds together? One obvious first step is to construct the descriptions that will be available for the PBI: *Solanum* project so that they can be searched using the emerging plant ontologies, or controlled vocabularies, enabling effective connection between these databases and quite disparate datasets [2,12]. We will also need to modify and expand these ontologies in order to encompass the full range of character diversity within *Solanum*. One can envisage, as a starting point, completing the circle in Figure 3 as a way of connecting data from genomics and biodiversity through the information-rich datasets of specimens and associated images generated by descriptive taxonomy. Another step is to connect the Solanaceae systematics and genomics communities by linking information and tools and establishing collaborative relationships to achieve a synergistic view of all aspects of Solanaceae biology.

One critical element for connecting these datasets in the future will be the preservation of voucher specimens for sequencing and for genetic analysis. A sequence of DNA does not belong to a species, it belongs to an individual (or at best a population); thus, for repeatability, voucher specimens should be made and deposited in publicly available collections (herbaria or museums). The lack of voucher specimens for what is a rather alarming amount of molecular work is at best sloppy science, but at worst positively dangerous (see examples in [21]). The data held in GenBank, while extremely useful, should be viewed with caution [9] unless accompanied by voucher specimen deposition information — 'most specimen data in GenBank are not congruent with potential repeatability of experiments' [21]. The specimen databases that will be made available through the *Solanum* PBI project will enable others to access this voucher information, will provide the impetus for voucher specimens to be routinely collected as part of both traditional and molecular work on the family. The PBI: *Solanum* project itself will bring together a cohort of experts skilled in specimen identification and curation as a resource for both the taxonomic and genomics communities, and will stimulate training in those traditional skills increasingly being seen as necessary for rigorous work in both fields. That the PBI: *Solanum* project will be set in a rigorous phylogenetic framework (see [1]) will strengthen and enhance the synergies created by linking these two fields and, we hope, will stimulate new approaches to the study of biodiversity in all its aspects.

Linking the traditions of museum science with those of the rapidly growing science of genomics will be the beginning of the real conversation between genomics and biodiversity — which will ultimately be of benefit to all, and will allow us to begin to answer the really big questions about life on Earth.

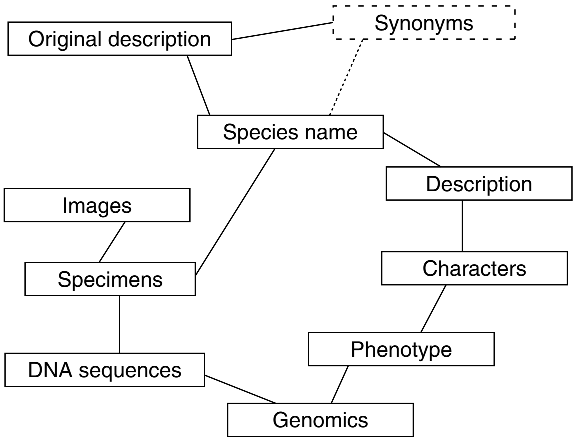

**Figure 3.** Linking taxonomy with genomics through description and specimens. Not all the elements of the two data types are included here; each synonym of an accepted name will have similar sets of data types associated with it. One critically important relationship seen here is that sequences and images are attached to specimens (individual plants), not to species names. The need for formal deposition of voucher specimens becomes clear when these relationships are examined closely. The description connected to the species name is based on the individual taxonomist's opinion of that species delimitation — specimens provide the validation of that opinion and are critical to maintaining data standards

### Acknowledgements

We thank Esther van der Knaap and Jiming Jiang for inviting S.K. to speak at the Solanaceae Workshop at PAG XII. The 'PBI: *Solanum* — a worldwide treatment' project is funded by NSF award DEB-01316614; we thank the reviewers and the committees for their support for descriptive taxonomy.

### References

1. Bohs L. 2004. Major clades in *Solanum* based in *ndhF* sequence analyses. In *Solanaceae: William G. D'Arcy*

Copyright © 2004 John Wiley & Sons, Ltd. 

*Memorial*, Hollowell V, Keating T, Lewis W, Croat T (eds). Monographs in Systematic Botany from the Missouri Botanical Garden (in press).

2. Bruskiewich R, Coe EH, Jaiswal P, *et al*. 2002. The Plant Ontology™ Consortium and plant ontologies. *Comp Funct Genom* **3**: 137–142.
3. Cnudde F, Moretti C, Porceddu A, Pezzotti M, Gerats T. 2003. Transcript profiling on developing *Petunia hybrida* floral organs. *Sex Plant Reprod* **16**: 77–85.
4. Daly DE, Cameron KM, Stephenson DW. 2001. Plant systematics in the age of genomics. *Plant Physiol* **127**: 1328–1333.
5. Dunal M-F. 1852. Solanaceae. In *Prodromus Systematis Naturalis Regni Vegetabilis* **13**(1). De Candolle ALPP (ed). Victor Masson: Paris; pp. 1–690.
6. Fay MF, Olmstead RG, Richardson JE, *et al*. 1998. Molecular data support the inclusion of *Duckeodendron cestroides* in Solanaceae. *Kew Bulletin* **53**: 203–212.
7. Gaston KJ, May RM. 1992. Taxonomy of taxonomists. *Nature* **356**: 281–282.
8. Godfray HCJ, Knapp S. 2004. Taxonomy for the twenty-first century. *Phil Trans R Soc B Biol Sci* **359**: (in press).
9. Harris DJ. 2003. Can you bank on GenBank? *Trends Ecol Evol* **18**: 317–319.
10. Heiser CB Jr. 1969. *Nightshades: The Paradoxical Plants*. W.H. Freeman: San Francisco, CA.
11. Hennig W. 1966. *Phylogenetic Systematics*. Wiley: New York.
12. Jaiswal P, Ware D, Ni J, *et al*. 2002. Gramene: development and integration of plant and trait ontologies for rice. *Comp Funct Genom* **3**: 132–136.
13. Kitching IJ, Forey PL, Humphries CJ, Williams DM. 1998. *Cladistics: The Theory and Practice of Parsimony Analysis*, 2nd edn. Oxford University Press: Oxford.
14. Knapp S. 2001. Is morphology dead in *Solanum* taxonomy? In *Solanaceae V: Advances in Taxonomy and Utilization*, Van den Berg RG, Barendse GWM, van der Weerden G, Mariani C (eds). Nijmegen University Press: Nijmegen; 23–38.
15. Knapp S. 2002a. Floral diversity and evolution in the Solanaceae. In *Developmental Genetics and Plant Evolution*, Cronk QCB, Bateman RM, Hawkins JA (eds). Taylor & Francis: London; 267–297.
16. Knapp S. 2002b. Tobacco to tomatoes: a phylogenetic perspective on fruit diversity in the Solanaceae. *J Exp Bot* **53**: 2001–2022.
17. Maes T, Van de Steene N, Zethof J, *et al*. 2001. *Petunia* Ap2-like genes and their role in flower and seed development. *Plant Cell* **13**: 229–244.
18. Olmstead RG, Palmer J. 1992. Chloroplast DNA phylogeny of the Solanaceae: subfamilial relationships and character evolution. *Ann Missouri Bot Gard* **79**: 346–360.
19. Olmstead RG, Sweere JA. 1994. Combining data in phylogenetic systematics: an empirical approach using three molecular datasets in the Solanaceae. *Syst Biol* **43**: 467–481.
20. Olmstead RG, Sweere JA, Spangler RE, Bohs L, Palmer J. 1999. Phylogeny and provisional classification of the Solanaceae based on chloroplast DNA. In *Solanaceae IV*, Nee M, Symon DE, Lester RN, Jessop JP (eds). Royal Botanic Gardens Kew: Richmond, Surrey; 111–117.
21. Ruedas LA, Salazar-Bravo J, Dragoo JW, Yates TL. 2000. The importance of being earnest: what, if anything constitutes a 'specimen examined'? *Mol Phylog Evol* **17**: 129–132.
22. Soltis DE, Soltis PA. 2003. The role of phylogenetics in comparative genetics. *Plant Physiol* **132**: 1790–1800.
23. Stebbins GL. 1974. *Flowering Plants: Evolution Above the Species Level*. Belknap; Cambridge, MA.
24. Symon DE. 1991. Gondwanan elements of the Solanaceae. In *Solanaceae III: Taxonomy–Chemistry–Evolution*, Hawkes JG, Lester RN, Nee M, Estrada N (eds). Royal Botanic Gardens Kew: Richmond, Surrey; 139–150.
25. Vandenbussche M, Zethof J, Souer E, *et al*. 2003. Toward the analysis of the petunia MADS box gene family by reverse and forward transposon insertion mutagenesis approaches: B, C and D floral organ identity functions require SEPALLATA-like MADS box genes in petunia. *Plant Cell* **15**: 2680–2693.

Copyright © 2004 John Wiley & Sons, Ltd. 

## References

[PDF_00423] Daly D. C., Cameron K. M., Stevenson D. W. (2001). Plant systematics in the age of genomics.. Plant Physiol.

[PDF_00440] Jaiswal Pankaj, Ware Doreen, Ni Junjian, Chang Kuan, Zhao Wei, Schmidt Steven, Pan Xiaokang, Clark Kenneth, Teytelman Leonid, Cartinhour Samuel, Stein Lincoln, McCouch Susan (2002). Gramene: development and integration of trait and gene ontologies for rice.. Comp Funct Genomics.

[PDF_00454] Knapp Sandra (2002). Tobacco to tomatoes: a phylogenetic perspective on fruit diversity in the Solanaceae.. J Exp Bot.

[PDF_00457] Maes T., Van de Steene N., Zethof J., Karimi M., D'Hauw M., Mares G., Van Montagu M., Gerats T. (2001). Petunia Ap2-like genes and their role in flower and seed development.. Plant Cell.

[PDF_00418] Plant Ontology Consortium (2002). The Plant Ontology Consortium and plant ontologies.. Comp Funct Genomics.

[PDF_00471] Ruedas L. A., Salazar-Bravo J., Dragoo J. W., Yates T. L. (2000). The importance of being earnest: what, if anything, constitutes a "specimen examined?".. Mol Phylogenet Evol.

[PDF_00474] Soltis Douglas E., Soltis Pamela S. (2003). The role of phylogenetics in comparative genetics.. Plant Physiol.

[PDF_00482] Vandenbussche Michiel, Zethof Jan, Souer Erik, Koes Ronald, Tornielli Giovanni B., Pezzotti Mario, Ferrario Silvia, Angenent Gerco C., Gerats Tom (2003). Toward the analysis of the petunia MADS box gene family by reverse and forward transposon insertion mutagenesis approaches: B, C, and D floral organ identity functions require SEPALLATA-like MADS box genes in petunia.. Plant Cell.

